# The “Little-Old-Lady's Hernia”, Obturator Hernia: A Case Report and Literature Review

**DOI:** 10.1155/2024/1039438

**Published:** 2024-06-18

**Authors:** Enrique Salazar-Rios, Alexa Cruz Olascoaga

**Affiliations:** ^1^ División de Estudios de Posgrado Facultad de Medicina Universidad Nacional Autónoma de México, México City, Mexico; ^2^ Departamento de Gastrocirugía Hospital de Especialidades “Dr. Bernardo Sepúlveda Gutiérrez” Centro Médico Nacional Siglo XXI Instituto Mexicano del Seguro Social, México City, Mexico; ^3^ Unidad Médica de Alta Especialidad Hospital de Gineco Obstetricia No. 3 “Dr. Víctor Manuel Espinosa de los Reyes Sánchez” Centro Médico Nacional la Raza Instituto Mexicano del Seguro Social, México City, Mexico

## Abstract

Obturator hernias, though rare, are clinically significant abdominal hernias, predominantly affecting elderly, thin women, with an estimated prevalence of less than 1%. The primary treatment involves surgical intervention to reduce and repair the defect, either through laparotomy or laparoscopy, with bowel resection needed in up to 75% of patients. Here, we present the case of an 83-year-old woman presenting with abdominal pain and a history of constipation. An abdominal computed tomography scan demonstrated a left obturator hernia with small bowel obstruction. Successful reduction of the hernia was achieved, albeit requiring intestinal resection via an open surgical approach. Subsequently, the patient achieved complete recovery.

## 1. Introduction

Obturator hernias represent a rare yet clinically significant form of abdominal hernias, accounting for less than 1% of all abdominal wall defects [1, 2, 3]. They are commonly known as the “little-old-lady's hernia” because they are observed more frequently in elderly, thin women [4]. Various risk factors have been observed to contribute to the development of these types of hernias, including emaciation, advanced age, and malnutrition [5]. Clinically, obturator hernias often manifest with nonspecific symptoms, including abdominal pain, distension, nausea, vomiting, and constipation [2]. The resemblance of these symptoms to those of more common gastrointestinal conditions makes diagnosis challenging. Consequently, imaging studies such as computed tomography (CT) are often essential to confirm the diagnosis. Management of obturator hernias typically entails surgical intervention, with the primary objective being a reduction of the herniated bowel and subsequent repair of the hernia defect [6, 7]. Timely diagnosis and intervention are crucial to prevent adverse outcomes given the high risk of complications, such as strangulation and necrosis [8].

## 2. Case Presentation

An 83-year-old female patient was admitted to the hospital with complaints of abdominal pain alongside a 3-day history of constipation. Upon admission, her body mass index was calculated to be 16 kg/m^2^. Relevant medical history included diagnoses of type 2 diabetes and systemic hypertension. She had previously experienced intermittent abdominal distension, nausea, vomiting of gastric contents, and poor oral intake. Following initial evaluation by a primary care physician, she was subsequently referred to our hospital for surgical evaluation after the placement of a nasogastric tube.

Physical examination revealed an elderly female with an emaciated appearance and poor hydration of the oral mucosa. The abdomen was distended with abolished peristalsis and was painful upon palpation in all quadrants, though rebound tenderness was absent. Inguinal or leg pain was interrogated and denied by the patient. No visible or palpable hernias were found, and no abnormal masses were detected on palpation. Rectal examination revealed an empty rectal ampulla without evidence of abnormal masses. The extremities were thin, with reduced muscle mass.

Initial blood tests (Table 1) revealed mild neutrophilia without leucocytosis, as well as mild hyponatremia and hypochloremia, likely due to vomiting and poor oral intake. The tests also indicated hypoalbuminemia, related to the patient's deficient nutritional state. Kidney function was preserved, with serum creatinine at 0.76 mg/dL. Blood gas analysis demonstrated metabolic acidosis without hyperlactatemia, likely due to the patient's dehydration.

**Figure 1:**
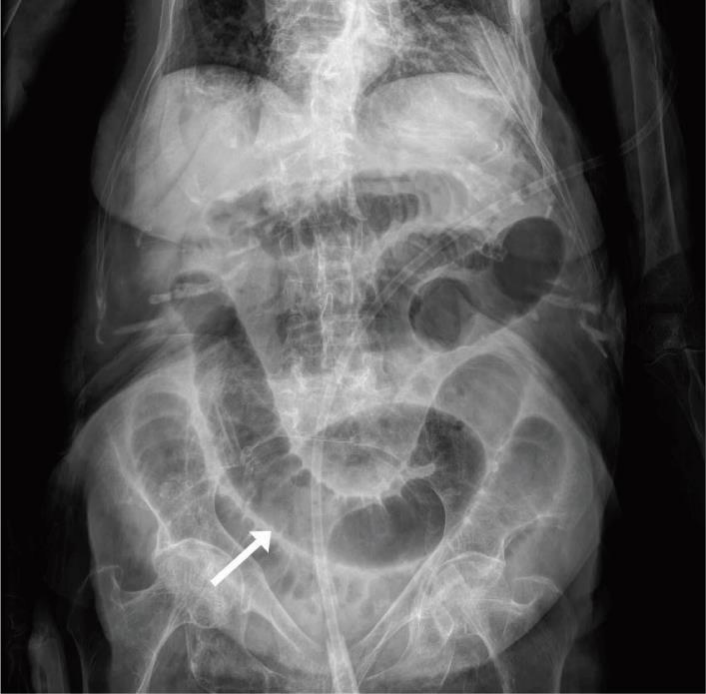
Plain abdominal X-ray demonstrating dilated loops of small bowel (white arrow), with marked valvulae conniventes and absence of gas in the rectal ampulla.

The initial abdominal X-ray is shown in Figure 1. Small bowel dilation (up to 40 mm, normal 25–30 mm) [9, 10] is observed in all quadrants, along with the absence of gas in the rectal ampulla.

**Figure 2:**
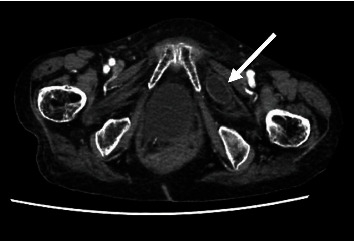
Axial contrast-enhanced computed tomography: representative image demonstrating a segment of small bowel protruding through the obturator canal (white arrow).

**Figure 3:**
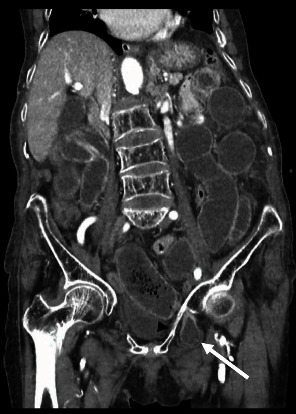
Coronal reconstruction of contrast-enchanted computed tomography: representative image showing a segment of small bowel protruding (white arrow) underneath the superior pubic ramus (black arrowhead) consistent with an obturator hernia. The rest of the small bowel is seen dilated up to 40 mm.

Clinical and radiologic findings, along with the history of constipation, suggested small bowel obstruction. Given the patient's absence of prior surgical history, a contrast-enhanced CT scan was performed to determine the etiology of the obstruction. The CT scan demonstrated dilated segments of the small intestine, most prominent in the proximal ileum up to 40 mm with the intestinal wall thickened, measuring up to 4.6 mm. A section of small intestine passing between the left internal and external obturator muscles was observed, indicative of an obturator hernia, with a defect measuring 6.7 mm, forming a sac measuring 32 × 21 mm in its anteroposterior and transverse dimensions (Figures 2 and 3).

An emergency laparotomy was performed based on the diagnosis of obturator hernia. Approximately 60 mL of reactive fluid was aspirated, and further exploration revealed a segment of the ileal wall (approximately 120 cm from the ileocecal valve) herniated and incarcerated at the left obturator; with the defect measuring less than 1 cm wide. Dilation of the small bowel was evident proximal to the herniated site, accompanied by wall edema and congestion. The herniated intestinal segment was successfully reduced, revealing a 5 cm ischemic zone that failed to regain vitality after reduction. Therefore, a 20 cm segment of the small intestine, including the ischemic portion, was resected, and a lateral-to-lateral anastomosis of the small bowel was performed. Primary repair of the hernia was conducted using absorbable suture.

The patient remained hospitalized, receiving intravenous rehydration and antibiotics. The patient showed favorable progress, with oral intake resumed on the third postoperative day and was discharged after a successful recovery at the fifth postoperative day. On follow-up at 4 weeks, the patient continued to progress favorably, with only a mild seroma present upon examination, which was successfully drained. At 2 months, the patient had made a full recovery and was discharged from our service.

## 3. Discussion

Obturator hernias are a rare subset of abdominal hernias, accounting for less than 1% of all abdominal wall hernias, and are responsible for approximately 0.2%–1.6%, of cases involving mechanical small bowel obstruction [1, 2, 3]. However, the majority of patients affected by these types of hernias suffer from small bowel obstruction, with more than 90% of patients exhibiting this symptomatology [2].

Despite their rarity, obturator hernias are associated with significant morbidity and mortality, particularly when complications, such as strangulation arise. These types of complications increase mortality drastically, reaching rates ranging from 12% to 70%, making these types of hernias the most lethal of abdominal wall hernias [11, 12]. Important predicting factors for mortality include old age, preexisting pulmonary conditions, preoperative hematocrit, serum sodium, serum creatinine and serum urea levels, and the absence of bowel motion [8]. These factors directly correlate to the patient's hemodynamic status, and their inflammatory systemic response, which is a key part in the preoperative management of these patients. Thus, optimizing a patient's hemodynamic status by addressing preoperative resuscitation is crucial for improving mortality.

Obturator hernias tend to be more prevalent in elderly women, particularly those who are slender, with females comprising more than 97% of affected patients [4]. The incidence rate is disproportional compared with men, being up to nine times higher [1]. Additionally, they are more frequently seen between the seventh and ninth decades [13]. Several anatomical factors can explain the gender and age susceptibility observed in this demographic. Elderly women tend to be thinner, due to aging-related changes, and have a wider pelvis. Additionally, their obturator canal opening tends to be more triangular with a greater transverse diameter, which facilitates herniation [2]. Another significant risk factor contributing toward obturator hernias is emaciation, a common characteristic among elderly individuals. The loss of preperitoneal fat and lymphatic tissue of the corpus adiposum over the obturator canal reduces protective padding and thus increasing the susceptibility to herniation [1]. This is particularly frequent in elderly patients with malnutrition, adding to the greater incidence observed in this demographic [2, 6]. Moreover, various underlying medical conditions can exacerbate the risk of obturator hernia by increasing intraabdominal pressure. Chronic obstructive pulmonary disease, chronic constipation, and ascites are notable examples of such, each contributing to elevated abdominal pressure, and facilitating herniation [5]. Older age, lower body mass index, and female gender have also been associated with a higher risk of bowel obstruction in patients with obturator hernia [14].

Interestingly, obturator hernias are twice as commonly observed on the right side [15]. This asymmetry is attributed to the anatomical location of the sigmoid colon, which predominantly occupies the left side of the pelvis which may lead to increased pressure and vulnerability on the right side, predisposing individuals to herniation in that specific area [5, 7, 11]. Interestingly, our patient presented with a left-sided obturator hernia.

The formation of obturator hernias typically occurs in three anatomical stages, which have been well described in the literature. Understanding the anatomy of the obturator region is crucial to comprehend the pathophysiology of obturator hernias.

The obturator region is located within the medial aspect of the upper thigh. It is bounded by the superior pubic ramus above, the hip joint and femur shaft laterally, the pubic arch, perineum, and gracilis muscle medially, and the origin of the adductor magnus below. Within this region lie the origins of the adductor muscles, the obturator canal, and the obturator foramen, the largest in the body, formed by the ischium and pubis rami [16]. Most of the obturator foramen is covered by the obturator membrane, with only a small opening remaining uncovered at its superior-lateral part, marking the beginning of the obturator canal. This canal, measuring 2–3 cm in length and 1 cm in width, extends downward and medially from the pelvis into the thigh. Within it, reside the obturator nerve, artery, vein, and a fat cushion called the corpus adiposum [16, 17]. The herniation itself takes place through the obturator foramen, along the obturator canal.

Initially, preperitoneal connective tissue and fat enter the pelvic orifice of the obturator canal, forming a plug. Subsequently, the peritoneum dimples into this fatty plug over the internal opening of the obturator canal, progressing to the invagination of a peritoneal sac. Finally, the hernia sac is occupied by intra-abdominal viscera, most commonly the small bowel, producing the symptoms associated with an obturator hernia [5, 16]. The contents of the hernia vary, although the most common ones include the small bowel, colon, omentum, and, in women, the ovary or fallopian tube [17].

The signs and symptoms of obturator hernia are generally nonspecific, and physical examination is often inconclusive because of the anatomical location of the hernia. Typically, patients afflicted present with symptoms of intestinal obstruction, such as nausea, vomiting, and abdominal cramps [2, 6]. An important diagnostic hallmark is the Howship–Romberg sign, which results from the compression of the obturator nerve by the hernia sac within the canal [6]. This compression leads to medial thigh pain that exacerbates with actions that increase abdominal pressure, such as coughing, hip flexion, external rotation, and thigh abduction [18]. This sign can be identified in nearly half of patients [2]. Although it is considered a pathognomonic sign, its use is limited as it can be easily mistaken for joint pain prevalent in the elderly population. Another important diagnostic sign is the Hannington–Kiff sign, consisting of the disappearance of the adductor reflex of the affected thigh as a result of obturator nerve compression [18].

Obturator hernias can also manifest as intermittent abdominal pain in up to 75% of patients [13]. This is attributed to Richter hernias, a type of partial bowel wall herniation which often spontaneously reduces resulting in temporary alleviation of the symptoms. Richter hernias are observed in a higher proportion (41%–100%) of obturator hernias [5]. Despite these symptoms, the hernia itself is not palpable or easily detectable through physical examination due to its deep location, thus requiring a high degree of clinical suspicion to consider this diagnosis. In our case, the patient presented with intermittent signs of small bowel obstruction prompting her to seek initial medical consultation, as well as abdominal pain and distention. However, the Howship–Romberg sign was not present in our patient.

Due to the nonspecific nature of its presentation and the limited clinical findings associated with obturator hernias, radiological imaging plays a critical role in the preoperative diagnosis of this pathology. Usually, the initial imaging modalities obtained include plain abdominal X-rays, as they are fast, accessible, and cost-effective. These X-rays are capable of demonstrating small bowel dilatation and air-fluid levels, suggestive of small bowel obstruction [2].

Ultrasound can play a significant role in the radiological diagnosis of obturator hernias, as it offers a noninvasive, radiation-free diagnostic tool. It is generally faster and can detect herniation, level of obstruction, presence of peristalsis, and vascular compromise of the affected intestinal segment; however, its efficacy is operator-dependent, as it involves a region with limited clinical application, which may lead to a lack of familiarity [19, 20]. Therefore, adequate training and expertise are essential for correct interpretation. Particularly, in lower income regions where more expensive studies such as CT scans are not accessible, ultrasound represents an important diagnostic tool.

Abdominopelvic CT is considered the gold standard for imaging diagnosis, as scans have shown a high level of sensitivity (up to 80%) in detecting obturator hernias [7, 11]. Importantly, it can diagnose up to 90% of cases before surgery thus helping in surgical planning [21]. It can also give indirect evidence of strangulation when distended small bowel loops or air-fluid levels are present, or an abrupt narrowing of the affected section is observed [11, 18]. Additionally, CT scans can aid in diagnosing small bowel obstruction. A retrospective study found that hernia sac diameter and volume were an independent risk factor for small bowel obstruction [14]. Unfortunately, despite facilitating a definitive diagnosis of obturator hernia, CT scans have not resulted in a reduction of bowel strangulation rates or postoperative morbidity and mortality [2]. In our case, the prompt use of contrast-enhanced CT scan allowed for timely diagnosis by observing the protrusion of small bowel through the obturator canal, the dilation of the rest of the small bowel, and wall edema of the affected segment.

The diagnostic challenges provided by obturator hernias often result in delayed treatment. Manual hernia reduction has been reported in certain instances; however, it is typically reserved for patients considered unsuitable for surgery [22]. Prompt operative repair is crucial for obturator hernias due to the high risk of bowel incarceration and strangulation, which often necessitates bowel resection, if left untreated, procedures associated with high mortality rates [6]. Reported bowel resection rates range from 25% to 75%, with mortality rates reported between 15% and 50% [7]. Various surgical approaches, including abdominal, inguinal, retropubic, obturator, and laparoscopic methods, have been described. Among these, the abdominal approach, typically executed using a low midline incision, is the most frequently used [6]. Advantages of a midline incision include extensive access to the abdominal cavity, facilitating hernia reduction, repair, and bowel resection when necessary, and providing versatility in cases with uncertain preoperative diagnoses [3]. As for surgical repair techniques, these vary, including primary peritoneal closure with stitches, reconstruction using tissue flap, and the use of surgical mesh, aiming to achieve tension-free closure [5]. Out of these, a primary suture repair with absorbable stitches is the most common open approach and is usually attempted for defects measuring less than 1 cm in diameter [3, 20]. Our patient underwent an emergency laparotomy, and due to the size of the defect, a primary closure with absorbable sutures was undertaken.

Recurrence is an important metric to assess hernia repair success. Overall, the open approach has exhibited a reported recurrence rate between 10% and 16% [3, 23]. However, a recent meta-analysis conducted by Burla et al. [4] revealed that the use of mesh in hernia repair significantly reduces the recurrence rate. Only 2% of patients who underwent mesh repair experienced hernia recurrence, compared to a 10% recurrence rate in those who underwent primary closure [4]. Traditionally, concerns regarding the risk of mesh infection have constrained its use in the setting of a contaminated surgical field. However, recent data have challenged this idea, revealing no statistical difference in postoperative complications or morbidity between patients treated with mesh and those without, with only a marginally increased risk of postoperative infection rates in the mesh group [4, 24, 25]. In a series conducted by Karasaki et al. [23], five patients underwent obturator hernia repair with mesh after requiring small bowel resection, all of whom showed no postoperative complications highlighting the safety and effectiveness of mesh use even in contaminated scenarios.

With advancements in laparoscopy, laparoscopic repairs, including transabdominal preperitoneal and totally extraperitoneal approaches, have also been performed. Although feasible for small bowel obstruction and obturator hernia, the use of laparoscopy is still somewhat controversial. The choice between laparotomy and laparoscopy for obturator hernia repair is widely debated, but usually involves patient condition a surgeon experience. The main limitations of the laparoscopic approach are a limited abdominal cavity due to the dilated small bowel and the requirement of greater surgical expertise to carry out the procedure. However, laparoscopic repair has some advantages over open procedures. It has been associated with less bleeding, a shorter postoperative length of stay, and fewer postoperative complications compared with an open approach [7, 15, 26]. Specifically, one study by Schizas et al. [5] demonstrated that laparoscopic repair was associated with a significantly reduced postoperative morbidity rate, with less than 10% of cases requiring conversion to laparotomy. Additionally, the laparoscopic approach allows for a comprehensive assessment of the groin region, specifically the myopectineal orifice, enabling the identification of other potentially occult groin hernias [3]. Some studies have shown that approximately 16% of patients who undergo laparoscopic repair of a diagnosed hernia are diagnosed and treated for additional, unexpected hernias, with up to 7% of these being undiagnosed obturator hernias [12, 27, 28]. The use of laparoscopy prevents the patient from undergoing reoperation and its associated comorbidities. Thus, laparoscopy represents a viable and often better option when dealing with obturator hernias. Unfortunately, laparoscopy is not always available, especially in developing countries. In such cases, open surgery remains the standard approach for obturator hernia repair. This was the situation in our case, where the patient presented during the night shift when laparoscopy was not available, and priority was given to prompt surgical intervention. Consequently, the patient underwent an open procedure. Despite this, a successful repair was achieved.

## 4. Conclusion

Obturator hernias represent a rare clinical entity that predominantly affects elderly, thin women because they are at an increased risk of emaciation, low body mass index, and conditions that raise intra-abdominal pressure. Diagnosis is challenging due to nonspecific symptoms like intermittent abdominal pain and distention which is why radiological imaging is crucial in obtaining a preoperative diagnosis and surgical planning. CT scans are considered the gold standard, but ultrasound is an option where CT is unavailable. Early surgical repair is key to avoid complications. Both open and laparoscopic approaches are used, with laparoscopy showing better outcomes but limited availability. Importantly, mesh use in defects over 1 cm, previously discouraged due to infection risk, reduces recurrence without significantly increasing infection risk. In our case, early recognition and intervention led to a successful recovery of the patient, despite the need for intestinal resection.

## Figures and Tables

**Table 1:** Blood exams taken upon the patient's admission.

Laboratory test	Result	Normal range
Leukocyte count	6.76 × 10³/*μ*L	4.6–10.2 × 10³/*μ*L
Absolute neutrophils	5.27 × 10³/*μ*L (77.9%)	1.5–7.0 × 10³/*μ*L (50%–70%)
Serum sodium	133.1 mEq/L	135–145 mEq/L
Serum chloride	96.1 mEq/L	98–106 mEq/L
Serum albumin	3.3 g/dL	3.5–5.0 g/dL
Serum creatinine	0.76 mg/dL	0.57–1.11 mg/dL
pH	7.29	7.35–7.45
pCO_2_	47 mmHg	35–45 mmHg
pO_2_	266 mmHg	80–100 mmHg
Lactate	1.3 mmol/L	0.5–2.2 mmol/L
HCO₃	22.6 mmol/L	22–28 mmol/L
Base excess (BE)	−4.2 mmol/L	−2 to +2 mmol/L

## Data Availability

All clinical data and images regarding this case report and supporting its findings have been included in the article.

## Abbreviations

CT: Computer tomography.
